# *Mycobacterium marinum* infection in an older woman with extensive verrucous skin lesions: a case report

**DOI:** 10.1590/S1678-9946202567031

**Published:** 2025-05-26

**Authors:** Hongyuan Shao, Yongxia Liu, Donghong Du, Hongqing Tian

**Affiliations:** 1 Shandong First Medical University, Hospital for Skin Diseases, Shandong, Jinan, China; 2 Shandong Academy of Medical Sciences, Shandong Provincial Institute of Dermatology and Venereology, Shandong, Jinan, China

**Keywords:** Mycobacterium marinum, Fish injury, Quantitative polymerase chain reaction sequencing technology

## Abstract

*Mycobacterium marinum* is a nontuberculous mycobacterium and an opportunistic pathogen which infects humans. Here, we report a case of an 87-year-old female who had extensive verrucous skin lesions on her upper limbs for 18 months. The patient was diagnosed with *Mycobacterium marinum* infection by pathology and quantitative polymerase chain reaction (qPCR) assay of skin tissue. After five months of oral rifampicin and clarithromycin, the skin lesions regressed completely.

## INTRODUCTION

The incidence of *Mycobacterium marinum* infection is growing. Recently, many cases of this infection have been reported, including several outbreaks. For instance, a retrospective study in the United States revealed that the infection incidence increased from 0.7 to 2 per 100,000 person-years from 1980 to 1999 and 2000 to 2009^1^. In Denmark, the incidence rate was reported to be 0.04–0.06 per 100,000 person-years during 2004–2009, which rose to 0.05–0.13 per 100,000 person-years from 2010 to 2016^2^. A report from China also indicated the same upward trend in the incidence of *Mycobacterium marinum* infection^3^.

The clinical manifestations are highly diverse, challenging both diagnosis and treatment for clinicians. Here we report a case of an older woman with multiple and slow-growing extensive verrucous skin lesions.

## CASE REPORT

**Figure 1:**
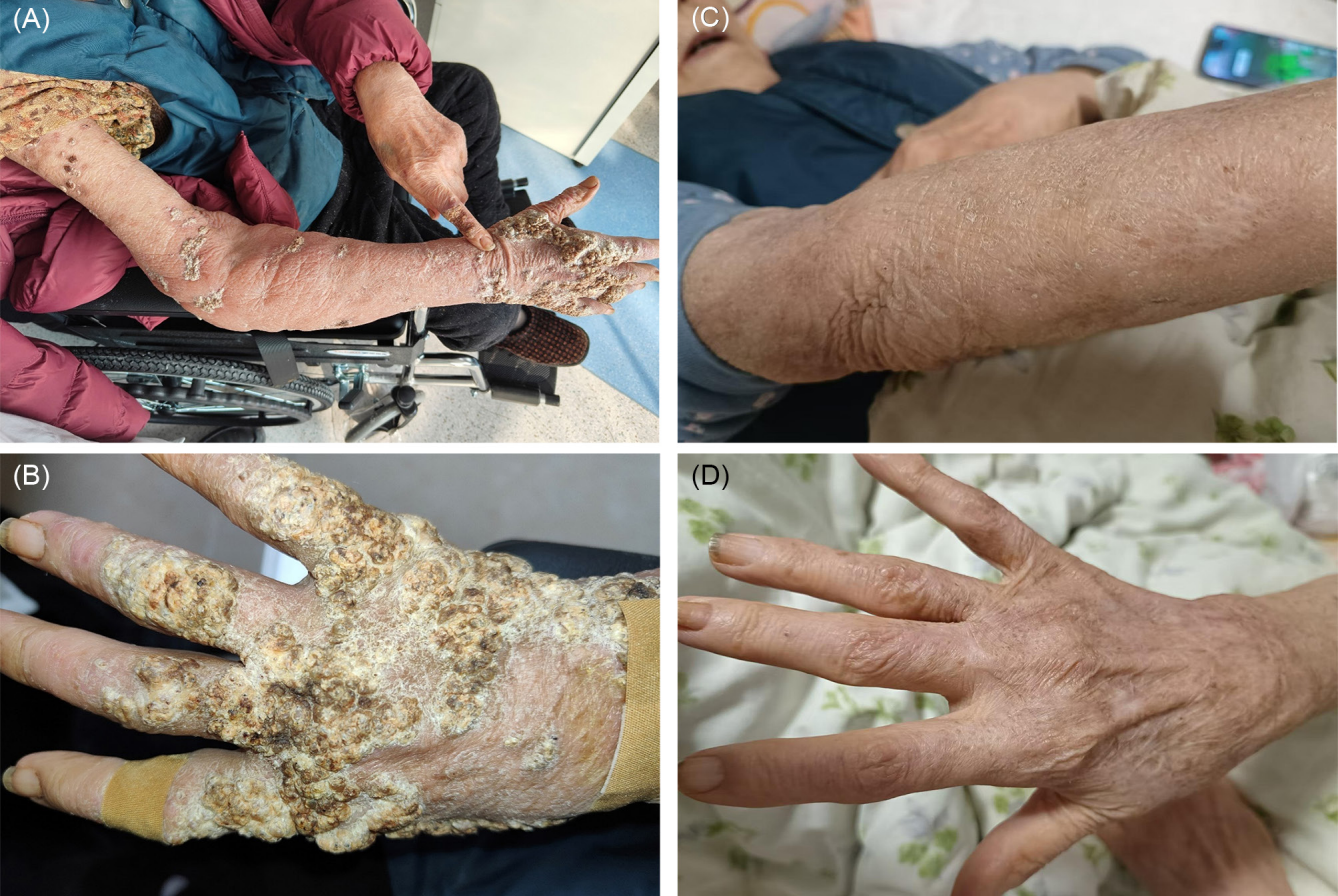
(A-B) Baseline: verrucous plaques on the right hand and scattered verrucous skin lesions on the left hand and right upper limb, with no enlarged lymph nodes; (C-D) After five months of treatment, all verrucous skin lesions regressed completely, leaving hyperpigmentation without obvious scarring.

**Figure 2:**
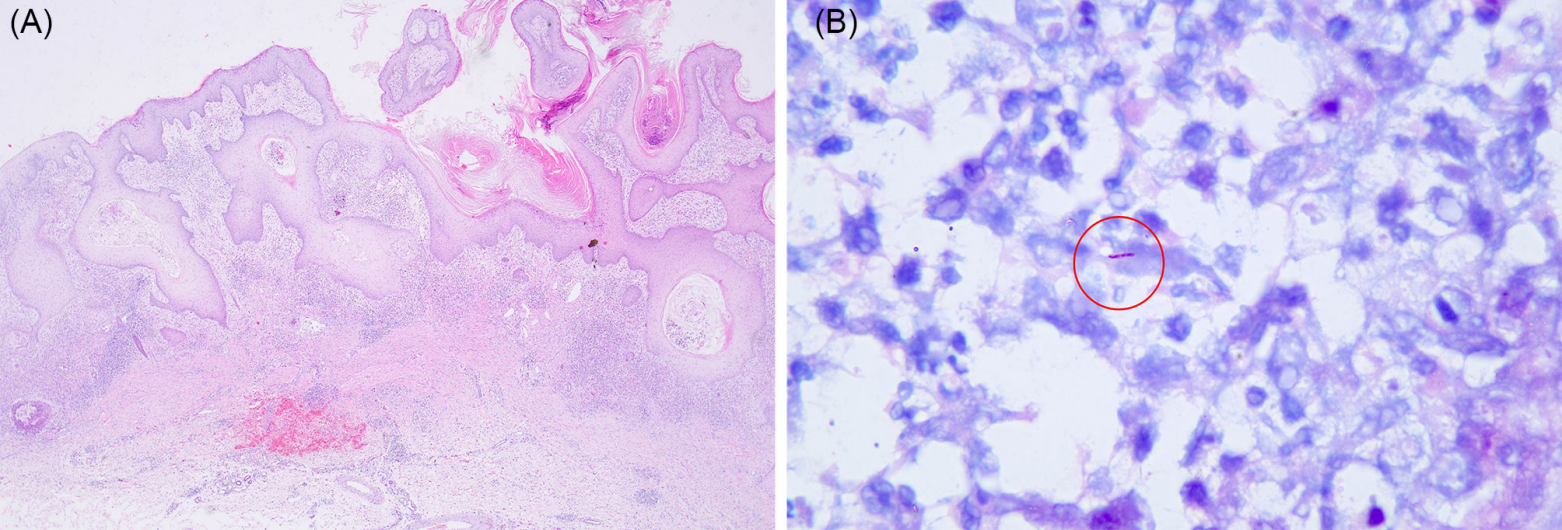
(A) Histopathological examination showed hyperkeratosis, parakeratosis, and irregular hyperplasia, with infiltration of lymphocytes, histiocytes, multinucleated giant cells, neutrophils, plasma cells in the superficial dermis, and proliferation of collagen fibers. (H&E, original magnification ×100); (B) Acid-fast staining: the position marked by the red circle is acid-fast bacilli. (original magnification ×400).

The patient was an 87-year-old female who had extensive verrucous skin lesions on the upper limbs for 18 months. She recalled that the lesions appeared two months after being pricked by a fish bone. In the beginning, multiple verrucous skin lesions gradually appeared on the back of her right hand and the right arm. The lesion slowly extended to the left hand 12 months later. The patient presented no lymphadenopathy, no fever, or any other systemic symptoms. A physical examination revealed the existence of verrucous plaques on the back of the right hand and scattered verrucous skin lesions on the right arm and left hand, with no enlarged lymph nodes (Figures 1A and 1B). The patient had a prior diagnosis of diabetes, nephropathy, heart failure, coronary heart disease, and cerebral infarction. Histopathological examination showed hyperkeratosis, parakeratosis and irregular hyperplasia of epidermis, and infiltration of lymphocytes, histiocytes, multinucleated giant cells, neutrophils, plasma cells in the superficial dermis, and proliferation of collagen fibers (Figure 2A). Acid-fast staining showed acid-fast bacteria (Figure 2B). The quantitative polymerase chain reaction (qPCR) technique, culture, and drug sensitivity tests yielded positive results for *Mycobacterium marinum*.

### qPCR methodology

The primers used for qPCR were F: CCGATGCCGATC TTGACTTC and R: AGGTCGTGCCAGTCGTTGTC. Probe sequences used for qPCR were *Mycobacterium marinum*-probe FAM-CTTCGGTGGACCGCTGA-MGB. The qPCR reactions were conducted using a TaqMan Universal PCR Master Mix (Applied Biosystems, USA) on a StepOnePlus Real-Time PCR System (Applied Biosystems, USA). The cycling conditions were as follows: 50 °C for 2 min and 95 °C for 10 min, followed by 40 cycles of 95 °C for 15 s and 60 °C for 1 min. A positive control (*Mycobacterium* DNA) and a negative control (water) were included in each run. The threshold cycle (Ct) values were recorded and analyzed. A sample was considered positive for *M. marinum* infection if the Ct value was less than 35.

After confirming *Mycobacterium marinum* infection, the patient was treated with oral rifampicin and clarithromycin for five months. All the verrucous skin lesions faded away, leaving hyperpigmentation without visible scarring. (Figures 1C and 1D).

## DISCUSSION

*Mycobacterium marinum* is a nontuberculous mycobacterium (NTM), that occurs ubiquitously in the human environment as an opportunistic pathogen. It induces infection in fish, causing a disease in humans via traumatized skin. The infection occurs after a traumatic injury and concurrent or subsequent exposure to contaminated water or infected fish^4^. Systemic dissemination of this infection is rare and has only been reported in immunocompromised patients.

The *Mycobacterium marinum* infection may be classified into four clinical categories (type I–IV). Type I, in immunocompetent patients, is known as single or limited (one to three) lesions marked by superficial cutaneous infection, appearing in the forms of crusted or ulcerated nodules or verrucous plaques. Type II, *Mycobacterium marinum* infection erupts as numerous (more than three) lesions with inflammatory nodules or in a sporotrichoid spreading pattern, or with abscesses and granulomas in an immunosuppressed patient and can progress to nodular lymphangitis. Type III of *Mycobacterium marinum* infection appears as deep infections associated with or without skin infection, the symptoms include arthritis, tenosynovitis, osteomyelitis, and/or bursitis. Type IV of the *Mycobacterium marinum* infection is disseminated via lung disease and other systemic manifestations^5^. In our case, the patient had a clear history of fish injury and, combined with the signs and auxiliary examination, it was classified as type II infection.

## CONCLUSION

Skin lesions of non-tuberculous mycobacteria are often misdiagnosed. *Mycobacterium marinum* infection is a diagnosis that must be considered as a differential diagnosis with verrucous tuberculosis, sporotrichosis, chromomycosis, squamous cell carcinoma, as the growth of *Mycobacterium marinum* is slow, delaying the diagnosis and treatment. As our case shows, the patient suffered for 18 months and had extensive verrucous skin lesions on the upper limbs with a considerable delay in the diagnosis.

Currently, the clinical treatment for *Mycobacterium marinum* cutaneous infection includes antibiotics such as ethambutol, linezolid, tetracycline, clarithromycin, moxifloxacin, or rifampicin^6^ and surgery. New methods such as cryotherapy, photodynamic therapy (ALA-PDT), and fractional laser have shown promising results and can be considered as alternative treatments when the traditional ones cannot be used or have poor efficacy^7^.
